# Health Effects of Ambient Air Pollution in Developing Countries

**DOI:** 10.3390/ijerph14091048

**Published:** 2017-09-12

**Authors:** Pier Mannuccio Mannucci, Massimo Franchini

**Affiliations:** 1 Scientific Direction, Fondazione IRCCS Ca’ Granda-Ospedale Maggiore Policlinico and University of Milan, 20100 Milan, Italy; 2 Department of Transfusion Medicine and Hematology, Carlo Poma Hospital, 46100 Mantova, Italy; massimo.franchini@asst-mantova.it

**Keywords:** air pollution, health, developing countries, low-income, children

## Abstract

The deleterious effects of ambient air pollution on human health have been consistently documented by many epidemiologic studies worldwide, and it has been calculated that globally at least seven million deaths are annually attributable to the effects of air pollution. The major air pollutants emitted into the atmosphere by a number of natural processes and human activities include nitrogen oxides, volatile organic compounds, and particulate matter. In addition to the poor ambient air quality, there is increasing evidence that indoor air pollution also poses a serious threat to human health, especially in low-income countries that still use biomass fuels as an energy resource. This review summarizes the current knowledge on ambient air pollution in financially deprived populations.

## 1. Introduction

It is well known that air pollution has a number of detrimental effects on human health and is considered a major issue for the global community [1,2,3,4,5,6]. The World Health Organization (WHO) estimated that, in the year 2012, ambient air pollution was responsible for nearly seven million deaths, representing more than 10% of all-cause deaths and more than doubling previous estimates [7,8]. Air pollution accounts worldwide for an estimated 9% of deaths due to lung cancer, 17% due to chronic obstructive pulmonary disease, more than 30% due to ischemic heart disease and stroke, and 9% due to respiratory infections [7]. The Global Burden of Disease report [9] identified in 2012 that air pollution was among the leading risk factors for disease burden, being globally responsible alone for 3.1% of all Disability-Adjusted Life Years (DALYs). All these findings confirm that air pollution is now the world’s largest environmental health risk.

Outdoor air pollution is a mixture of thousands of components. Among them, airborne particulate matter (PM) and the gaseous pollutants ozone, nitrogen dioxide (NO_2_), volatile organic compounds (including benzene), carbon monoxide (CO), and sulphur dioxide (SO_2_) are the most important from a health perspective. Primary pollutants such as soot particles and oxides of nitrogen and sulphur are emitted directly into the air by the combustion of fossil fuels [10]. Major sources of primary particles include motorized road traffic, power generation, industrial sources, and residential heating. Secondary pollutants, formed when primary pollutants react or interact in the atmosphere, include mainly ozone (O_3_) and PM [11]. The latter, primary or secondary in origin, consists of particles that, on the basis of their size, are classified as coarse (diameter < 10 μm; PM_10_), fine (diameter < 2.5 μm; PM_2.5_), or ultrafine (<0.1 μm; PM_0.1_). PM_2.5_ are contained within the coarse particle fraction, broadly representing approximately 50% of the total mass of PM_10_. The resuspension of soil and road dust by wind or moving vehicles, as well as construction work and industrial emissions, results in coarse particles (PM_10_). Fine particles are derived primarily from direct emissions from combustion processes such as gasoline and diesel fuel, wood burning, coal burning for power generation, and industrial processes [11]. Fine particles can travel large distances (more than 100 km), with the potential for high background concentrations over a wide area [12]. As a consequence, their composition may be extremely heterogeneous, depending on the meteorological conditions and human activities in a particular geographical area [12]. Ultrafine particles are fresh emissions from combustion-related sources such as vehicle exhaust and atmospheric photochemical reactions and are recognized as important markers of exposure to traffic exhaust along main roads [13]. Fine and ultrafine particles are those associated with the worst effects on health as they can reach the deepest portions of the airways or even reach the blood stream directly [5,14]. 

If acute and long-term exposure to ambient air pollution represents a serious threat for health in western industrialized countries, the burden of this problem is even higher in developing countries, where population explosion along with widespread industrialization coupled with urbanization have resulted in dense urban centers with poor air quality [15,16]. In such developing countries, however, huge economic and social disparities coexist; thus, in addition to the poor ambient air quality, people can be also exposed, especially in rural areas, to high concentrations of indoor air pollution due to the use of biomass fuels (coal, wood, and other solid fuels) as an energy resource [17,18]. Worldwide, more than three billion people, largely in developing countries, rely on biomass fuels for their domestic energy needs [8]. As a consequence, household air pollution from solid fuel use has become a serious threat to health and has been estimated to be one of the top five major risk factors for the global burden of disease (4.3% of global DALYs), accounting for 3.9 million premature deaths in 2010 [17]. In this regard, the best example is provided by Asia, which has experienced rapid and disharmonic industrialization, urbanization, and transportation development in the recent decades, with resulting outdoor and indoor air pollution levels that are constantly well above the upper limits indicated by the WHO guidelines [19,20]. China, in particular, the Asian country with the fastest industrial development and population increase, is now facing the worst air pollution problem in the world [21,22]. This review focuses on this particularly vulnerable population living in low- and middle-income countries and highly exposed to both household and outdoor pollutants.

## 2. Health Effects in Adult Population

A number of studies and meta-analyses have shown that increased mortality is associated with short- and long-term exposure to PM, both in developed and developing countries. The percentage relative risk (RR) increase for all-cause mortality related to short-term PM exposure has been estimated to range from 0.4% to 1.5% per 20 μg/m^3^ increase in coarser PM_10_ and from 0.6% to 1.2% per 10 μg/m^3^ increase in finer PM_2.5_ [23]. Interestingly, the results of a recent large study conducted in the United States by Di and colleagues [24], including more than 60 million Medicare beneficiaries from 2000 through 2012, found that, for every increase of 10 μg/m^3^ in PM_2.5_, there was an associated 7.3% increase in all-cause mortality. This robust association was even more evident when the analysis was restricted to the Medicaid-eligible sub-group, documenting that persons with low socioeconomic status are more likely to be exposed to higher pollutant levels (and thus experience an increased incidence of adverse effects) than the rest of population [24]. As previously mentioned, this association may particularly important in East-Asian countries, which, due to their rapidly developing economies and dense populations, are exposed to very high levels of air pollution [25]. For instance, with urbanization increasing from 26% in 1990 to 50% in 2010, China has undergone dramatic epidemiological transitions [26,27], and, among the risk factors responsible for DALYs, ambient and household air pollution ranked fourth and fifth, respectively [28]. A recent meta-analysis of 33 time-series and case-crossover studies conducted in China to assess the mortality effects of short-term exposure to air pollution observed that each 10 μg/m^3^ increase in PM_2.5_ was associated with a 0.38% (95% CI 0.31–0.45) increase in total mortality, a 0.51% increase in respiratory mortality (95% CI 0.30–0.73), and a 0.44% (95% CI 0.33–0.54) increase in cardiovascular mortality [25]. The short-term effects of air pollution on health have also been the object of intense research in developing countries outside the Asiatic region. For instance, the ESCALA (Estudio de Salud y Contaminacion del Aire en Latinoamerica) study, which was conducted in nine Latin American cities, found a significant association between daily exposure to PM_10_ and O_3_ and daily mortality [29]. Likewise, indoor air pollution has been shown to have a significant impact on the health of populations living in rural areas in less industrialized countries such as Pakistan and India [30,31].

Pertaining to the adverse effects on the lungs, air pollution, as previously mentioned, is the cause and aggravating factor of many respiratory diseases like chronic obstructive pulmonary disease, asthma, and lung cancer [32]. Increased ambient O_3_, NO_2_, PM_2.5_, and SO_2_ levels were consistently associated with increased hospital admission for asthma and pneumonia in various studies conducted in Hong Kong and Taipei [33,34,35,36]. A systematic review confirmed that indoor air pollution due to solid fuel combustion was also an important risk factor for chronic obstructive pulmonary disease in adult populations living in low-income countries, particularly in non-smoking women [37]. In addition, a number of studies have consistently documented the association between air pollution and the risk of developing lung cancer; women carry the highest risk, probably due to their increased exposure to indoor air pollution [38,39,40]. Time-based multiple risk factor models have shown that smoking and solid-fuel use collectively contributed to 75% of lung cancer deaths in China [41]. Each 10 μg/m^3^ increase in the two-year average of PM_2.5_ correlated significantly with an increased risk of lung cancer in both males (RR 1.055; 95% CI 1.038 to 1.072) and females (RR 1.149; 95% CI 1.120 to 1.178) [42].

In addition to respiratory diseases, there is also increasing evidence that sustained exposure to ambient and household air pollution has a particularly deleterious effect on the cardiovascular system, and an association has been found between hypertension, coronary heart disease, and stroke [16]. A 2013 review reported a pooled effect of 11% (95% CI 6 to 16%) for cardiovascular mortality with a 10 μg/m^3^ PM_2.5_ increase [43]. Although populations in low- and middle-income countries are highly exposed to environmental pollution, the bulk of evidence that links these exposures to cardiovascular disease is derived mostly from populations in high-income countries [16]. Pertaining to developing countries, for instance, the results of a recent study conducted by Chen and colleagues [44] clearly indicate that life-expectancy in Northern China, where air quality is particularly poor, is 5.5 years lower owing to an increased incidence of cardiorespiratory mortality. Notably, a study on long-term exposure to ambient air pollution conducted in Shenyang, the largest and most heavily industrialized city in China, showed that an increase of 10 μg/m^3^ in the yearly average concentration of PM_10_ corresponded to 55% and 49% increases in the risk of death due to cardiovascular and cerebrovascular diseases, respectively [45]. In addition to the close link between air pollution and arterial thrombosis, there is also some evidence of the association with venous thromboembolism [45], as suggested by a recent systematic review [46]. A study conducted in Santiago, Chile (a city with a high level of air pollution) between 2001 and 2005 reported that the short-term increase in hospital admissions for venous thrombosis and pulmonary events was proportional to the elevations in the concentration of fine PM, documenting that the burden of this phenomenon is significant also for developing countries [47].

## 3. Health Effects in Particularly Vulnerable Populations

If air pollution, both outdoor and indoor, is considered a major health problem in developing countries, the burden of this issue is even greater in those population groups that are particularly vulnerable such as pregnant women, newborns, and children [48,49]. In particular, exposure to indoor air pollution from the combustion of solid biofuels is a significant public health hazard predominantly affecting women and small children living in poor households in both rural and urban communities in developing countries [50]. Recent studies have shown that air pollution can affect the developing fetus via maternal exposure, resulting in preterm birth, low birth weight, growth restriction, and potentially adverse cardiovascular and respiratory outcomes [51]. In this regard, a number of epidemiological and clinical studies conducted in low-income countries found an association between exposure to indoor air pollution during pregnancy and low birth weight and still birth [52,53,54,55,56,57], and a meta-analysis by Pope and colleagues calculated that the RR of low birth weight and stillbirth attributable to indoor air pollution in developing countries was 21% and 26%, respectively [58]. Another more recent systematic review and meta-analysis found a strong association between household air pollution from solid fuel use and the risk of adverse pregnancy outcomes; such exposure resulted in an 86.43 g reduction in birth weight and a 35% and 29% increased risk of low birth weight and stillbirth, respectively [59]. Interventions aimed at reducing exposure to household air pollution will result in an improvement of survival outcomes for all children [19]. Notably, a study evaluating the mortality effects of indoor air pollution and ambient urban PM pollution in Mexico estimated that the annual child mortality rate would decrease by 0.1 per 1000 children in the absence of these environmental exposures [60]. 

There is also consistent evidence that smoke from biofuels can cause acute lower respiratory infections in childhood [61,62,63]. Notably, a randomized controlled trial performed in Guatemala found that a reduction in exposure to household air pollution led to a significant one-third reduction of severe childhood pneumonia, with possible important implications for the reduction of child mortality [64]. In addition, air pollution has been found to be an important contributor to the increased prevalence of allergic diseases in children in developing countries, including asthma [32,65,66]. Coal combustion for heating (odds ratio (OR) 1.5, 95% CI 1.1 to 1.9) and cooking (OR 2.3. 95% CI 1.5 to 3.5) conferred higher risks of childhood asthma in China [67]. Each 10 μg/m^3^ increase in NO_2_ corresponded to an adjusted OR of 1.25 (95% CI 1.16 to 1.36) for diagnosed asthma in six to 13 year-old Chinese children [68]. In addition, increased ambient O_3_, NO_2_, PM_2.5_, and SO_2_ levels were associated with increased hospital admission for asthma in children [33,34,35,69,70]. For instance, Ko and colleagues [34] retrospectively reviewed the relationship between daily emergency hospital admissions and asthma and indices of air pollutants (SO_2_, NO_2_, O_3_, PM_10_, and PM_2.5_ levels) for 15 major hospitals in Hong Kong between January 2000 and December 2005. A total of 69,716 admissions were assessed, and significant associations were found between hospital admissions for asthma and NO_2_, O_3_, PM_10_, and PM_2.5_ levels (respective RRs, 1.04, 1.03, 1.02, and 1.02 per 10 μg/m^3^ increases). However, the adverse effects of early-life exposure to air pollution could be long lasting. In China, each 15 μg/m^3^ and 50 μg/m^3^ increase in NO_2_ and SO_2_ levels was associated with an adjusted OR of 1.90 (95% CI 1.20 to 3.00) and 1.62 (95% CI 1.01 to 2.60) for asthma, respectively, whereas the combined exposure to high levels of NO_2_ and SO_2_ further increased the OR to 1.85 (95% CI 1.22 to 2.79) in the first year of life [71].

## 4. Conclusions

Compared with most developed countries that have completed industrialization programs for several years, low- and middle-income countries have experienced an intense process of urbanization and industrial development in a very short period of time, which has led them to become the countries with the largest air pollution-related burdens in recent years. This phenomenon has deleterious effects on the health of people resident in these developing countries, as they are exposed to the joint toxic effects of household and ambient air pollution (Table 1).

In particular, women and children living in severe poverty have the greatest exposure to indoor air pollution from solid fuel use as they spend a lot of time near stoves [72]. As a consequence, such vulnerable populations have an increased risk of developing short-term and long-lasting adverse effects related to air pollution and thus need a closer follow-up. In this regard, the systematic monitoring of ambient air quality by national authorities in such countries will enable the implementation and evaluation of interventions aimed at lowering dangerous air pollutant levels [19,73]. The expenditures of these programs will be largely amortized by the costs saved by the prevention of air pollution-related morbidity and mortality in the population [20]. An impressive example of the potential beneficial effects of such interventions on the health of the population stems from the Beijing 2008 Olympic Games, when maintaining the exposure to PM_10_ under the limit of 100 μg/m^3^ during the period of the Games was associated with a nearly 40% reduction in health-related economic costs, compared with the period before the Games [21]. In conclusion, further epidemiological and clinical studies on air pollution in the developing world are warranted for assessing the degree of the burden of air pollution on health outcomes and for setting priorities in taking environmental local control measures.

## Figures and Tables

**Table 1:** The burden of air pollution in developing versus developed countries.

Issue	References
Higher household exposures due to biomass fuels	[8,18,22,30,49,61]
More rapid and disharmonic industrialization, urbanization and transportation development	[8,19,22,26,28]
Fewer preventative health services and generally less surveillance	[18,19,73]
Higher maternal exposure during pregnancy	[52,53,56,57,58,59]
Higher children exposure	[66,67,68,69,70,71]
